# The Callosal Relay Model of Interhemispheric Communication: New Evidence from Effective Connectivity Analysis

**DOI:** 10.1007/s10548-017-0583-x

**Published:** 2017-08-12

**Authors:** Saskia Steinmann, Jan Meier, Guido Nolte, Andreas K. Engel, Gregor Leicht, Christoph Mulert

**Affiliations:** 1 Psychiatry Neuroimaging Branch, Department of Psychiatry and Psychotherapy, University Medical Center Hamburg-Eppendorf, Martinistr. 52, 20246 Hamburg, Germany; 2 Department of Neurophysiology and Pathophysiology, University Medical Center Hamburg-Eppendorf, Hamburg, Germany

**Keywords:** Auditory cortex, Dichotic listening task, Effective interhemispheric connectivity, EEG, Gamma-band oscillations

## Abstract

Interhemispheric auditory connectivity via the corpus callosum has been demonstrated to be important for normal speech processing. According to the callosal relay model, directed information flow from the right to the left auditory cortex has been suggested, but this has not yet been proven. For this purpose, 33 healthy participants were investigated with 64-channel EEG while performing the dichotic listening task in which two different consonant–vowel syllables were presented simultaneously to the left (LE) and right ear (RE). eLORETA source estimation was used to investigate the functional (lagged phase synchronization/LPS) and effective (isolated effective coherence/ICoh) connectivity between right and left primary (PAC) and secondary auditory cortices (SAC) in the gamma-band (30–100 Hz) during right and left ear reports. The major finding was a significantly increased effective connectivity in the gamma-band from the right to the left SAC during conscious perception of LE stimuli. In addition, effective and functional connectivity was significantly enhanced during LE as compared to RE reports. These findings give novel insight into transcallosal information transfer during auditory perception by showing that LE performance requires causal interhemispheric inputs from the right to the left auditory cortices, and that this interaction is mediated by synchronized gamma-band oscillations.

## Introduction

Interhemispheric auditory connectivity via the corpus callosum has been shown to be responsible for the timely interplay of right and left speech-relevant brain regions recruited for normal speech comprehension (Friederici et al. 2007). However, it remains largely unknown how the auditory systems dynamically interact with one another and in particular in which direction the interhemispheric communication is realized. According to the callosal relay and the left-hemispheric specialization for language and speech processing, the directed flow of information from the right to the left-dominant hemisphere during certain language tasks has been suggested, but not yet proven (Hugdahl and Westerhausen 2016). Effective connectivity (EC) analysis provides the next step concerning the understanding of callosal dynamics underlying auditory processing by examining causal information flow at the spectral nature of oscillatory activity between distinct predefined brain regions (Pascual-Marqui et al. 2014). With EC analysis, it is possible to create and test realistic models of interacting neuronal systems to investigate explicitly the directed influence of one region on another (Friston 2011). In particular, Granger causality analysis of electrophysiological (EEG) data offers the important advantage of high temporal resolution and the detailed investigation of specific frequency bands (Seth et al. 2015).

One of the most popular paradigms to investigate interhemispheric connectivity and hemispheric specialization of language and speech is the dichotic listening task. The term “dichotic listening” describes a paradigm in which two slightly different verbal stimuli (such as consonant–vowel syllables) are simultaneously presented, one to each ear, with the participants’ instruction to report the stimulus which was understood most clearly. Typically, the majority of healthy participants show the well-known right ear advantage (REA), that is, they report more often the right (RE) than the left ear (LE) stimuli (Hugdahl 2011). According to the “structural model” (Kimura 1967, 2011) or “callosal relay model” (Zaidel 1983), this REA is explained by the anatomy of the ascending auditory pathways and the left-hemispheric lateralization of language and speech processing (Hugdahl and Westerhausen 2016). Although the verbal stimuli can reach the auditory cortex via both contralateral and ipsilateral projections, it is assumed that under dichotic conditions the ipsilateral pathways are inhibited, while the contralateral pathways are more preponderant (Brancucci et al. 2004; Fujiki et al. 2002). Thus, only the right ear stimuli are directly transmitted to the relevant left-hemispheric processing areas, whereas the left ear stimuli—initially projected to the right hemisphere—require additional interhemispheric transfer across the corpus callosum in order to be finally processed in the speech-dominant left hemisphere. Accordingly, a “hardwired” buttom-up phenomenon seems to play a crucial role for the emergence of the REA. However, the magnitude of the REA also has been found to be associated with the structural and functional interhemispheric auditory connectivity: Using DTI-based tractography, it has been shown that there are remarkable shape differences among healthy individuals, with stronger fibers improving interhemispheric transfer so that participants reported more syllables presented to the left ear (Westerhausen et al. 2009). Moreover, using EEG recordings, evidence of our own group indicated that the functional connectivity (FC) between right and left secondary auditory cortices is mediated by synchronous gamma-band oscillations (GBO) (Steinmann et al. 2014a). Here, conscious perception of left ear syllables was significantly related to an increased interhemispheric gamma-band coupling, suggesting that GBO are a functional key mechanism in the transcallosal auditory transfer. However, the direction of information transfer during dichotic listening has not been investigated so far, although the callosal relay model suggests a clear direction.

Accordingly, it was the aim of this EEG study to investigate the relationship between functional and effective interhemispheric connectivity in the gamma-band (30–100 Hz) and lateralized auditory perception during dichotic listening. To address this question, eLORETA source estimation was used to determine (1) the FC using lagged phase synchronization (LPS), and (2) the EC using isolated effective coherence (ICoh) between right and left (and vice versa) primary (PAC) and secondary auditory cortices (SAC) in the gamma-band during conscious perception of either right or left ear syllables. Specifically, we hypothesized that the effective connectivity analysis proves that perception of left ear stimuli requires interhemispheric causal transfer in the gamma-band from the right to the left secondary auditory cortices, a finding that would be in accordance with the callosal relay model of dichotic listening.

## Methods

### Participants

The sample consists of 33 healthy right-handed German native speakers (18 male, 15 female). The participants’ handedness was verified with the empirically validated Edinburgh Handedness Inventory (Oldfield 1971). Exclusion criteria were left-handedness or a history of hearing, psychiatric or neurological disorders. To ensure normal hearing in both ears, all participants were screened with pure tone audiometry for frequencies between 125 and 8000 Hz (Esser Home Audiometer 2.0). Participants with an auditory threshold higher than 25 dB, or an interaural difference larger than 15 dB in any of the frequencies were excluded from the study. All participants had normal IQ as tested with a vocabulary test (Herzfeld 1994). The group of participants partly overlapped with the sample of our previous study (Steinmann et al. 2014a). This study was approved by the ethics commission of the Medical Association Hamburg (Reference number: PV3485). All applied methods were in accordance with all relevant guidelines and regulations. After participants received a complete description of the experimental procedures, written informed consent according to the Declaration of Helsinki was obtained. Demographic data for all participants are presented in Table 1.

**Table 1:** Demographic characteristics of the sample: mean, standard deviation (SD) and range are given for each variable

Variable	Demographic data of participants (n = 33)		
	Mean	SD	Range
Age (years)	31.36	9.11	19–57
Gender (male/female)	18/15	n.a.	n.a.
Handedness	87.74	16.27	40–100
Educational level	1.36	0.60	1 (high)–3 (low)
Verbal IQ	111.27	10.00	86–129
Laterality index (LI)	24.65	22.42	−16.84 to 67.00
Right ear reports	134.81 (56.17%)	30.64	79–192
Left ear reports	80.00 (33.33%)	21.72	37–123
Error reports	25.69 (10.70%)	17.18	5–66

### Paradigm

**Fig. 1:**
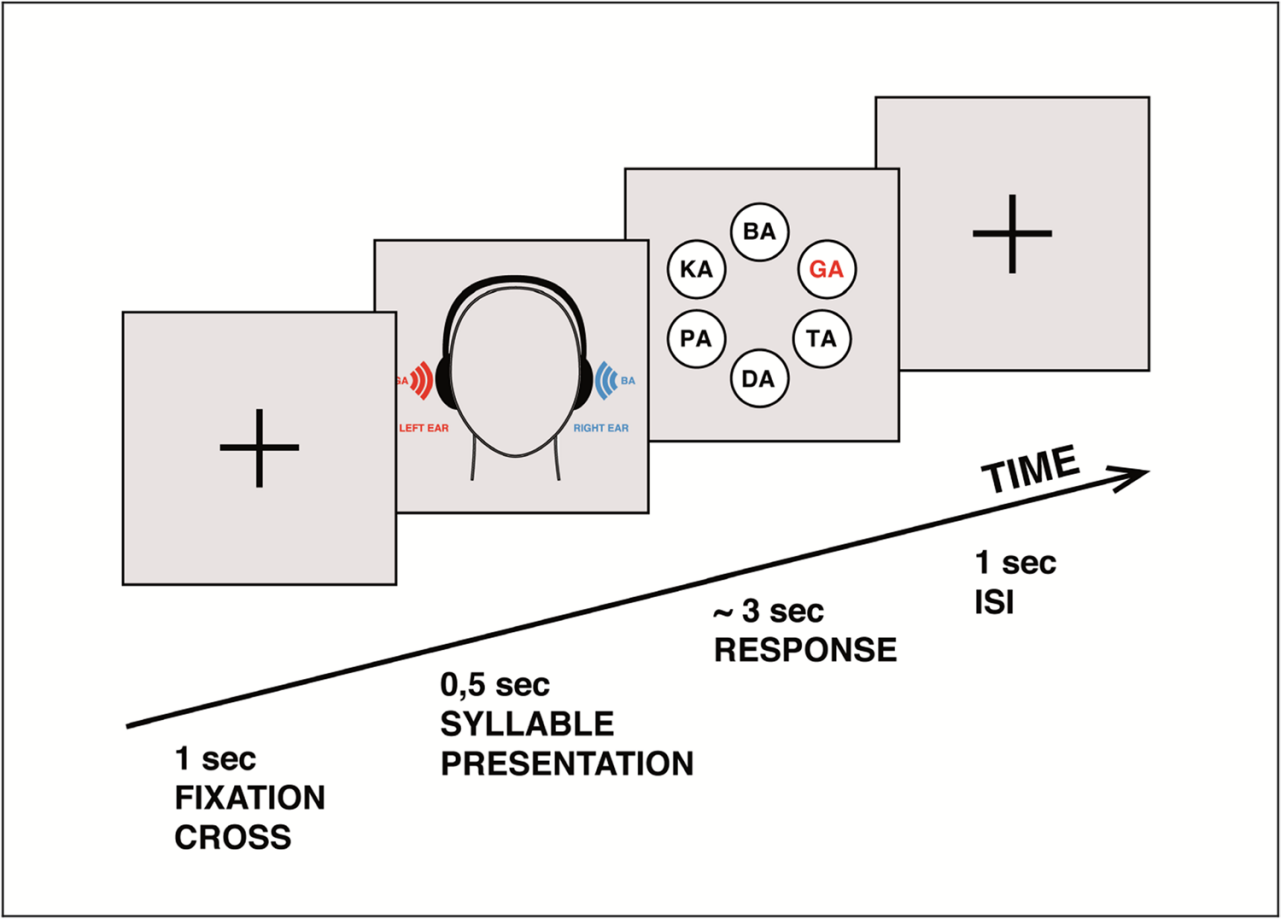
Dichotic listening task. The beginning of each trial was indicated by the appearance of a fixation cross in the centre of the screen. Subjects were instructed to fixate their eyes on the cross. The response was given via a response screen which appeared immediately after hearing the syllable pair. The screen showed all six syllables presented in a circular formation. By clicking with the* right* (dominant) hand the* left* mouse button it was possible to navigate through the six answer alternatives and with the right mouse button the selection was confirmed. Between the offset of the visual presentation and the onset of the next auditory stimulus a stable interstimulus interval (ISI) of 1 s was applied

The subjects had to perform a dichotic listening task that was also used in our previous study (Steinmann et al. 2014a). In brief, six different consonant–vowel syllables (/ba/, /da/, /ka/, /ga/, /pa/, /ta/) were paired and presented simultaneously with one syllable to each ear. In order to control effects of syllable voicing, only syllables with the same voice onset time (VOT) were combined, resulting in 12 possible dichotic pairs. VOT describes the length of time between the release of a consonant and the onset of voicing, defined by the vibration of the vocal folds. Three of the syllables were voiced (/ba/, /da/, /ga/) and had a short voice onset time (VOT) between 17 and 32 ms, and three were unvoiced (/pa/, /ta/, /ka/) with a long VOT in the range of 75–80 ms. Each syllable combination was temporally aligned to achieve simultaneous onset of the initial consonants. The mean duration was between 400 and 500 ms depending on the different VOT. After filling out the questionnaires and performing the hearing test, participants were asked to perform practice trials of 6 syllable pairs in order to get familiarized with stimulus material and experimental procedure. The main experiment consisted of 240 trials, which were presented in two blocks of 120 trials. Both blocks were presented to participants with the instruction to report the syllable they understood most clearly (non-forced condition), while they were not informed that each presentation consisted of two different syllables. Participants were encouraged to relax, reduce eye and head movement, fixate on the cross, and avoid jaw muscle contraction. Responses were made by button press using the dominant (right) hand. The stimulus administration and response collection were controlled using Presentation^®^ software (Neurobehavioral Systems, Albany, CA). After the recording, the percentage of correctly reported syllables was calculated separately for left and right ear stimuli. In order to assess the magnitude of the ear effect, a behavioral laterality index (LI) was calculated for every subject according to the formula: LI = 100 × (RE − LE)/(RE + LE); where RE = number of correct right ear reports and LE = number of correct left ear reports. The scale varies between −100 and +100, with negative values indicating a LEA and positive values a REA (Fig. 1).

### EEG Recording

The recording took place in a sound-proof and electrically shielded cabin, while participants listened through closed system headphones (Sennheiser, HAD 200) to the randomly presented 240 syllable pairs at approximately 75 dB. The EEG recordings were conducted at a sampling rate of 1000 Hz with 64 Ag/AgCl electrodes mounted on an elastic cap (ActiCaps, Brain Products, Munich, Germany) using the Brain Vision Recorder 1.10 (Brain Products, Munich, Germany). Eye movements were recorded using four EOG channels. Impedances were kept below 5 KΩ.

Offline processing was carried out using Brain Vision Analyzer 2.0 (Brain Products, Munich, Germany). The data was bandpass filtered from 20 to 120 Hz and down-sampled to 256 Hz. All channels were re-referenced to common average and FCz (reference during recording) was recovered as a regular channel. Epochs with muscle artifacts in any channel were identified by visual inspection and rejected from further analysis. Independent component analysis (ICA) was applied to identify and remove blink, horizontal eye movement, electrocardiographic, and saccadic spike potential (SP) artifacts based on their characteristic topographies, time-courses, and frequency distributions (Carl et al. 2012). In order to improve the identification of SP artifact components in the gamma-band frequency range (Yuval-Greenberg et al. 2008) an additional “radial electro-oculogram channel” (REOG, defined as the average of all EOG channels: REOG = (HEOGR + HEOGL + VEOGS + VEOGI)/4 − Pz.) was used following the procedure described by Keren et al. (2010). The SP artifact components appeared in the REOG channel consistently as a sharp biphasic deflection. Subsequently, the artifact-free data was segmented in epochs of 2048 ms starting 200 ms prior to stimulus onset. Correct-response epochs were exported for connectivity analysis and balanced between the number of right and left reports trials for each subject, ending up in a mean number of 82 trials for both conditions.

### Interhemispheric Auditory Connectivity Analysis

All further analyses were executed using the LORETA KEY software package as provided by Roberto Pascual-Marqui (The KEY Institute for Brain-Mind Research University Hospital Psychiatry, Zurich) at http://www.uzh.ch/keyinst/LORETA.html.

For analysis of FC, the lagged phase synchronization (LPS) between auditory cortices was calculated, as done previously (Steinmann et al. 2014a). LPS measures the similarity between signals in the frequency domain based on normalized (unit module) Fourier transforms; thus it is related to nonlinear functional connectivity (Pascual-Marqui et al. 2011). The LPS measure represents the connectivity between two signals after the instantaneous, zero-lag contribution has been excluded. Such a correction is necessary when using scalp EEG signals or estimated intracranial signals, because zero-lag connectivity in a given frequency band is often due to non-physiological effects or intrinsic physical artifacts, in particular volume conduction (Nolte et al. 2004; Stam et al. 2007). Thus, this measure removes this confounding factor considerably and is thought to contain only physiological connectivity information. The LPS formula is defined as:

1 $$\varphi _{{x \rightleftarrows y}}^{2}(t,\omega )=\frac{{{{\left\{ {\operatorname{Im} \left[ {{f_{x,y}}(t,\omega )} \right]} \right\}}^2}}}{{1 - {{\left\{ {\operatorname{Re} \left[ {{f_{x,y}}(t,\omega )} \right]} \right\}}^2}}}$$

where ***t*** denotes the time domain and ***ω*** denotes the frequency domain of the respective imaginary and real part from the complex coherency.

In order to account for the causal directionality at the spectral nature of oscillatory activity during dichotic listening, EC was computed as isolated effective coherence (iCoh) (Pascual-Marqui et al. 2014), where iCoh from region-of-interest (ROI) **j** to ROI **i** at a discrete frequency ***ω*** is defined as:

2 $${\upkappa _{i \leftarrow j}}(\upomega )=\frac{{[{{\text{S}}_\upvarepsilon }]_{{ii}}^{{ - 1}}{{\left| {{{\left[ {\overset{\lower0.5em\hbox{$\smash{\scriptscriptstyle\smile}$}}{\text{A}}(\upomega )} \right]}_{ij}}} \right|}^2}}}{{[{{\text{S}}_\upvarepsilon }]_{{ii}}^{{ - 1}}{{\left| {{{\left[ {\overset{\lower0.5em\hbox{$\smash{\scriptscriptstyle\smile}$}}{\text{A}}(\upomega )} \right]}_{ij}}} \right|}^2}+[{{\text{S}}_\upvarepsilon }]_{{jj}}^{{ - 1}}{{\left| {{{\left[ {\overset{\lower0.5em\hbox{$\smash{\scriptscriptstyle\smile}$}}{\text{A}}(\upomega )} \right]}_{jj}}} \right|}^2}}}$$

where **S(ɛ)−1** denotes the matrix inversion of the spectral density matrix (i.e., Hermitian covariance), and **A** denotes the autoregressive coefficients at a given frequency ***ω***, while resulting coherence values satisfy

3 $$0 \leqslant \upkappa {\text{i}} \leftarrow {\text{j}}\,(\omega ) \leqslant 1.$$

Contrary to the LPS analysis, this method provides the opportunity to assess the direct nature of neuronal connections under multivariate autoregressive (MVAR) modelling of partial directed coherence (Baccala and Sameshima 2001). Importantly, causal directionality between a priori defined ROI can only be estimated by setting the effects of all other possible neuronal connections to zero, which is a necessary condition in the assessment of Granger-causal influences (Granger 1969). In the present study, right and left primary auditory cortices (PACs/BA41), known to support any type of sound processing (Johnsrude et al. 2002), and right and left secondary auditory cortices (SACs/BA42), known to be involved in the processing of complex sounds and speech sounds (Binder et al. 2000; Zaehle et al. 2004), were defined as ROIs using the anatomical definitions provided by the eLORETA software based on the Talairach Daemon. Previously, we have reported LPS differences between right and left reports in two gamma sub-bands (slow gamma: 30–50 Hz, mid gamma: 50–90 Hz), but not in any other frequency band (delta, theta, alpha, beta). Therefore, LPS and iCoh analysis were focussed on the gamma-band range (30–100 Hz). In order to get high frequency resolution for the ICoh analysis, we decided for an AR-order of 8 (high order-concatenation), because the frequency resolution in linear AR modelling mainly depends on its order (Ding et al. 2000). Because MVAR modelling presupposes the issue of stationarity, we guaranteed synchronized trial data by epoching the exported segments to a shorter time window of 200 ms with an onset at 500 ms post-stimulus. The choice of this window was based on the fact that the syllable presentation ends around 500 ms, and importantly that non-directional connectivity between left and right SACs was found to reach highest synchrony during left ear report in this time window (Steinmann et al. 2014a). Thus, LPS and iCoh were calculated in a time frame from 500 to 700 ms to syllable presentation onset for right and left ear reports, respectively, and for iCoh in both directions (left to right hemisphere and vice versa, respectively). Under consideration of all randomized and re-epoched trials, one mean iCoh-value (frequency resolution: 1 Hz) was calculated for each subject and each direction. Finally, iCoh values in the gamma-band range were averaged from 30 to 100 Hz.

### Statistics

SPSS version 22 was used for the statistical analysis of behavioral and demographic characteristics (http://www.spss.com). For all analyses the significance level was set to α = 0.05. All data were tested for normality using the Kolmogorov–Smirnoff-test and for Sphericity using Mauchly’s test. In case of violation of the sphericity assumption, Greenhouse–Geisser-corrected *p*-values and degrees of freedom were reported. Effect sizes for significant results were quantified as η^2^-partial (RM-ANOVA) or r (Wilcoxon tests). A 2 × 2 repeated measures analysis of variance (RM-ANOVA) with *Condition* (RE/LE-reports) as within-subjects factor and *Gender* as between-subjects factor was used to check for gender differences with respect to the LI. Wilcoxon signed rank test was used to investigate differences in FC (LPS) in the gamma-band between *LE* and *RE Percepts* (for PAC and SAC respectively). For EC data (iCoh) we used two (for PAC and SAC respectively) 2 × 2 RM-ANOVAs with *Condition* (RE/LE-report) and *Direction* (Right to Left/Left to Right) as within-subjects factors. Significant main effects were further explored using Wilcoxon-signed ranks post-hoc tests which were corrected for multiple comparison with Bonferroni-holm.

## Results

### Task Performance

Participants reported significantly more syllables presented to the RE (135 ± 5.3) than to the LE (80 ± 3.8) as indicated by a main effect of *Condition* $[F_{1,32} = 40.93;~p < .001,~\eta ^{2} _{{partial}} = 0.57]$, reflecting the typical REA (LI: *M* = 24.65 ± 22.42). There was no significant main effect of *Gender* (p = .23) and and no significant *Condition × Gender* interaction (p = .23). The LI’s were normally distributed (one-sample Kolmogorov–Smirnov test, p = .74). 28 out of 33 participants showed a positive LI, whereas 4 participants had a negative LI and one subject showed no ear advantage (LI = zero).

### Interhemispheric Functional Connectivity (FC) Between Auditory Cortices

**Fig. 2:**
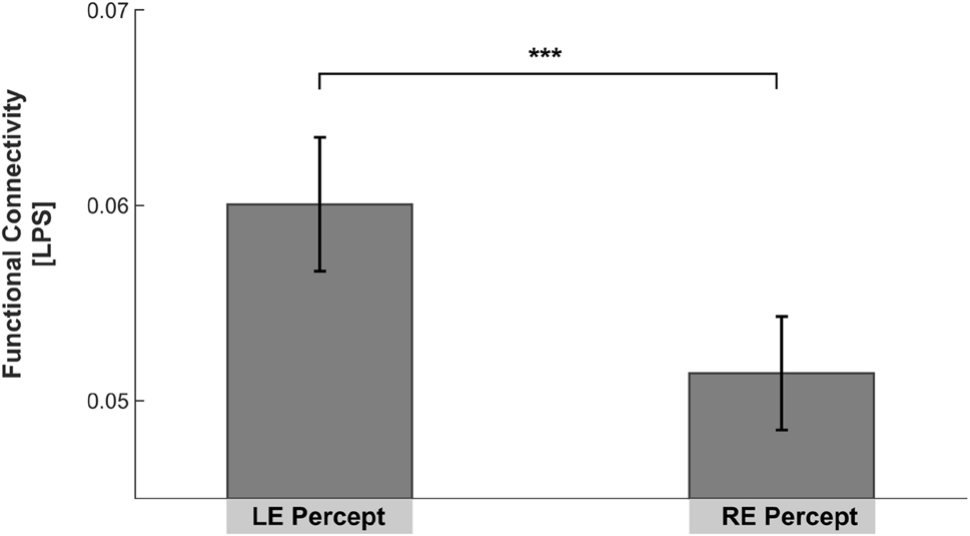
LPS between* right* and* left* SAC in the gamma-band frequency range (30–100 Hz) calculated for a time-window from 500 to 700 ms after stimulus onset. Significantly increased LPS was found during left ear (LE) compared to right ear (RE) Percept. Significant findings are highlighted with an *asterisk*

The Wilcoxon signed rank test revealed a significant increase of LPS between right and left SAC during *LE Percept* (*Md* = 0.0561) compared to *RE Percept* (*Md* = 0.0453) [*Z* = 3.181, *p* < .001, *r* = 0.55, Fig. 2]. There was no significant difference for PAC.

### Interhemispheric Effective Connectivity (EC) Between Auditory Cortices

**Fig. 3:**
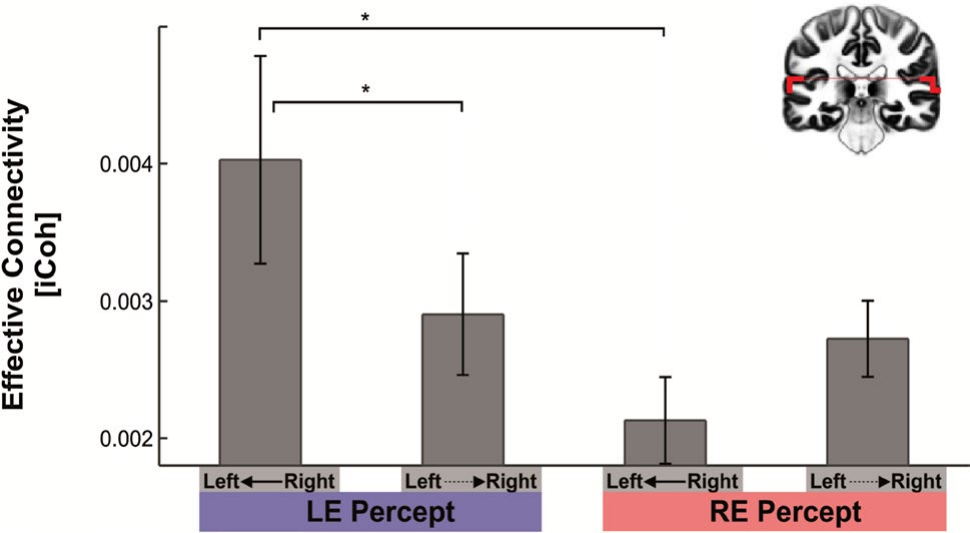
Means of iCoh of the four potential directions during left (LE) and right ear (RE) Percepts in the gamma-band frequency range (30–100 Hz): Significantly increased iCoh was found during LE Percepts (*blue*) from right to left SAC compared to the other direction (i.e., *left* to *right*). Moreover, significantly increased ICoh was found during LE Percepts (*blue*) compared to RE Percepts (*red*) for ICoh from right to left SAC. *Shaded error bars* represent 95% CI. Significant findings are highlighted with an *asterisk*. (Color figure online)

**Fig. 4:**
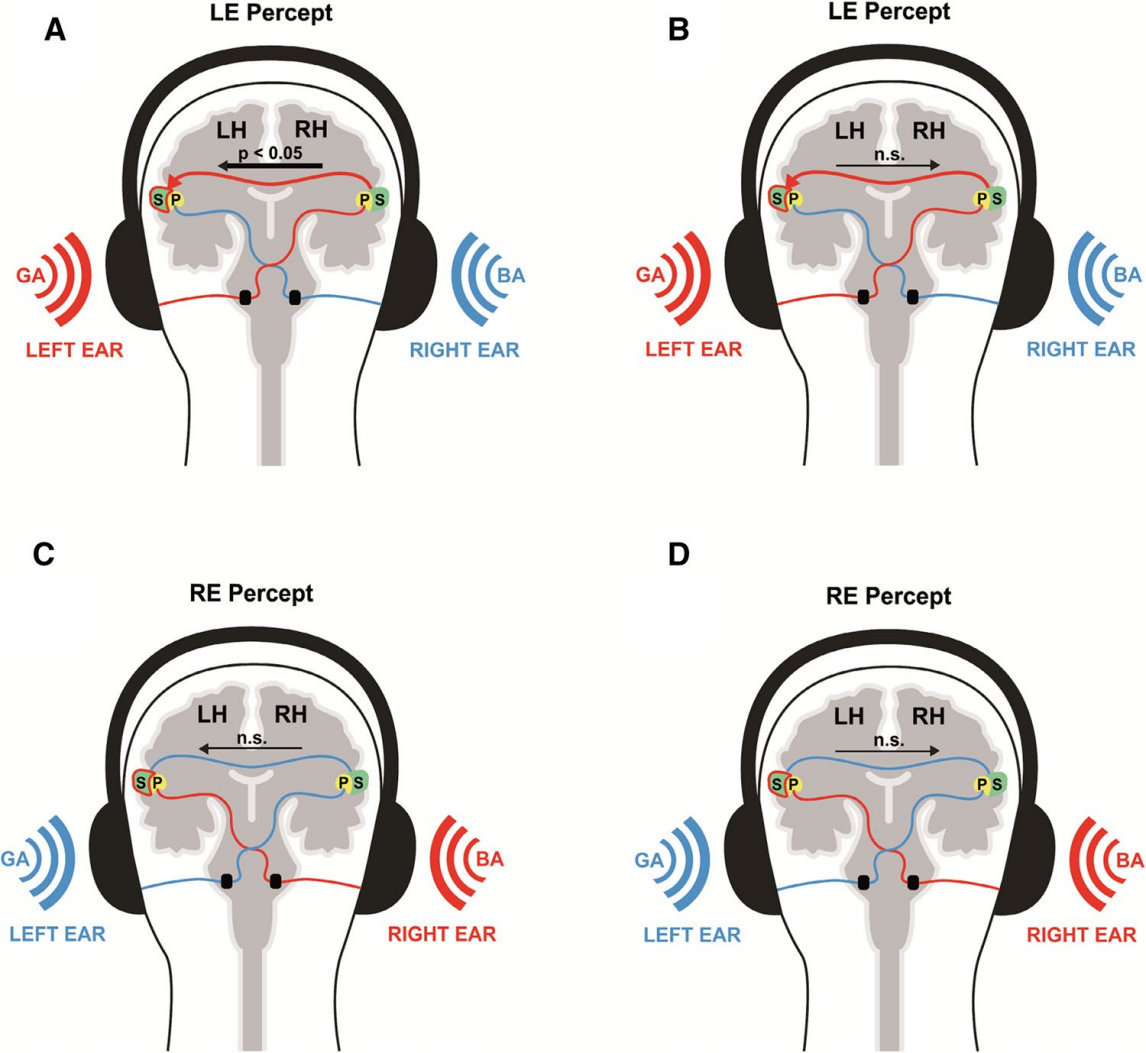
**A, B** Schematic illustration that displays the processing pathway underlying conscious perception of left ear (LE) syllables. The *thin red line* indicates the contralateral pathway transmitting the LE stimulus from the left ear directly to the non-dominant right hemisphere. The subsequent transfer from the right to the left SAC—which is assumed to be responsible for syllable analysis—is illustrated by the *thick red line*. ICoh analysis demonstrated that conscious perception of LE syllables is associated with increased information flow from the right to the left SAC (**A**), but not the other way round (**B**). **C, D** Schematic illustration that displays the contralateral processing pathway underlying conscious perception of right ear (RE) syllables, which does not require interhemispheric interaction. Conscious perception of RE syllables was not associated with a significant increased interhemispheric ICoh in any of the two directions. *LH* left hemisphere, *RH* right hemisphere, *P* primary auditory cortex, *S* secondary auditory cortex, *n.s*. not significant. (Color figure online)

In accordance to our hypothesis, there was a significant interaction effect of *Condition* ×* Direction* $[F_{1,32} = 6.666,~p = .014,~\eta ^{2} _{{partial}} = 0.17]$ for the whole gamma-band range. Bonferroni-holm corrected post-hoc tests revealed that the perception of syllables presented to the LE was accompagnied by a significantly increased interhemispheric ICoh from the right to the left SAC compared to the other direction (*Z* = 2.00, *p* = .025, *r* = 0.35; Fig. 3). Moreover, the iCoh from the right to the left SAC was significantly increased during perception of LE syllables compared to RE syllables (*Z* = 2.69, *p* = .016, *r* = 0.34), whereas the iCoh from the left to the right SAC showed no significant difference between LE and RE Percept. Perception of RE syllables was not accompanied by any significant difference between the two directions. There were no further significant main effects or an interaction effects, nor for any of the analyses for PAC (Fig. 4).

## Discussion

The aim of this study was to determine the degree and the direction of the interhemispheric auditory connectivity in the gamma-band by means of LPS and iCoh during dichotic listening and to further proof the concept of the callosal relay model. For this purpose, the dichotic listening is a well-suited paradigm, as it is one of the most frequently applied tasks for assessing language lateralization and interhemispheric interaction with a good understanding of the underlying structural substrate (Westerhausen and Hugdahl 2008).

The characteristic finding is the REA, which we were able to replicate in this study. In accordance with our hypothesis, the major finding was a significantly increased EC in the gamma-band from the right to the left SAC during conscious perception of left ear stimuli. In addition, this causal information flow as well as the gamma-band phase synchrony was significantly enhanced during LE as compared to RE reports. There was no significant difference between directions during conscious perception of RE syllables, indicating that this pathway is redundant. These results are fully consistent with the callosal relay model, suggesting that only left ear perception requires additional interhemispheric transfer from the right auditory cortex via the corpus callosum to the language-processing areas of the left hemisphere (Zaidel 1983). DTI-based tractography studies have shown that the splenium bordering the isthmus (both located at the posterior third of the corpus callosum) contains the interhemispheric pathways that interconnect primary and secondary auditory cortices (Hofer and Frahm 2006; Huang et al. 2005). This callosal region is characterized by large number of fast-conducting, highly myelinated auditory pathways of more than 3 µm in diameter (Aboitiz and Montiel 2003; Fabri and Polonara 2013). Thus, these fibers are the thickest among the callosal fibers suggested to promote synchronous activation across distant brain regions with high transmission velocity (Singer 1999). The understanding of hemispheric differences and interhemispheric interaction during dichotic listening was considerably improved through studies of split-brain patients (Springer and Gazzaniga 1975; Sugishita et al. 1995) and patients with splenial lesions (Pollmann et al. 2002) as well as patients with multiple scleroses (Gadea et al. 2002, 2009; Pelletier et al. 2001). Such studies demonstrated that atrophy or disruptions of the splenial commissures lead to enhanced REA or even a complete left ear extinction. All these data indicate that callosal disruptions impair the interhemispheric information transfer and alter the behavioural laterality index. Furthermore, in healthy participants a strong relationship between naturally occurring interindividual variability in midsagittal callosal area and the dichotic listening performance has been observed (Westerhausen et al. 2009; Yazgan et al. 1995). Here, a stronger interhemispheric connectivity resulted in a reduced REA, which is most likely caused by a better processing of the left ear stimuli. Thus, several pieces of evidence support the notion that conscious perception of left ear syllable requires interhemispheric interaction. Besides, the corpus callosum consists not only of homotopic but also heterotopic connections (Di Virgilio and Clarke 1997) and it might be speculated that the interhemispheric transfer from right to left could result from combined inputs of homotopic and heterotopic callosal fibers. In order to clarify this point, such EC analysis (i.e., from right PAC to left SAC and vice versa) were performed with no significant findings (all p > 0.5). This is in accordance with the literature suggesting that homotopic connections are more numerous (Jarbo et al. 2012) and exceptionally strong compared to heterotopic pathways (Shen et al. 2015).

To date, our knowledge of interhemispheric interaction during speech perception relies on the source space analysis of undirected statistical dependencies between ROIs (i.e., PAC and SAC) using LPS analysis as a tool of FC with high temporal resolution, emphasizing a crucial role of GBO for transcallosal functional coupling. The EC analysis provides now the next methodological step concerning the understanding of underlying causal mechanism of callosal transfer by indicating that this is mediated from right to left SAC. In contrast to FC, EC analysis offers the great advantage of permitting statements about directed statistical dependencies in a predefined neuronal system, comparing how well a defined model explains the observed data by performing a linear AR fitting in a specific order (Seth et al. 2015). Furthermore, EC is defined in both time and frequency domain and holds the potential to uncover the spectral characteristics of the measured interactions. In the present study, the main results suggest GBO to be the mechanism that coordinates the interhemispheric information flow from the right to the left SAC that subserve coherent auditory perception. This is of special interest, since a growing body of evidence already has indicated GBO and their synchronization as a fundamental mechanism that coordinates widely distributed neurons into dynamically formed functional networks that subserve coherent perception and cognition (Engel et al. 2001; Giraud and Poeppel 2012; Hipp et al. 2011). Interestingly, the structural and functional transcallosal connectivity has also been suggested to play a crucial role for the pathopyhsiolohy of auditory phantom percepts, such as auditory verbal hallucinations (AVH) in schizophrenia (Steinmann et al. 2014b). Here, disturbances of the interhemispheric auditory phase synchrony has been found again in the gamma-band frequency range (Mulert et al. 2011). Moreover, altered interhemispheric pathways (Mulert et al. 2012) as well as reduced language lateralization have been related to the emergence of AVH (Ocklenburg et al. 2013). Thus, to uncover the dynamical mechanism underlying the typical REA may not only be important for basic science on hemispheric lateralization and auditory perception, but may also have important implications for the understanding of clinical disturbances in such a network, as it can be observed in schizophrenia.

Concerning limitations and strengths of the present study that warrant discussion, the relatively low spatial accuracy has to be mentioned (Pascual-Marqui et al. 1994), although cross validation studies using simultaneous EEG and fMRI have suggested sufficient validity of the LORETA approach in general (Mulert et al. 2005, 2004). It has been shown that the Euclidean distance between EEG- and fMRI-based localizations typically ranges between 1 and 2 cm. However, our finding of increased LPS and iCoh between bilateral SACs is consistent with our previous EEG study that has evidenced the SAC to be the main contributor to the left ear report probability. At first sight, the application of an MVAR-model on times series of EEG signals may appear contradictory since this approach technically presumes the observed data as the outcome of a linear time-invariant system (Greenblatt et al. 2012), while the brain can clearly be considered as a non-linear system. Nevertheless, AR-modelling is a powerful tool to identify causal relationships in linear and non-linear domains of a predefined neural network, under careful consideration of issues like stationarity, temporal filtering and volume conduction (Nunez 1981). Furthermore, EC measures have to be regarded as complementary rather than competitive to other measures, such as FC, which provide even better frequency resolution. One promising methodological next step to elucidate the relation between functional, effective and structural connectivity might be the investigation with multimodal imaging, including the combination of EEG und fMRI (Mulert et al. 2010) or EEG and DTI (Lei et al. 2015) during dichotic listening. This study was not designed to clarify top-down attentional influences, which have been suggested to contribute to the emergence of the REA during dichotic listening tasks (Kinsbourne 1970; Kinsbourne and McMurray 1975). Here, further studies using EC analysis including conditions with attentional focus on either the left or the right ear would be helpful.

In sum, the EC findings give novel insight into transcallosal information transfer during auditory perception supporting the assumption that left ear performance requires causal interhemispheric transfer from the right to the left auditory cortices and that this interaction is mediated by synchronized GBO.
